# Change In Cycle Length During Narrow Complex Tachycardia: What Is The Mechanism?

**Published:** 2010-04-01

**Authors:** Diego Chemello, Anandaraja Subramanian, Douglas Ing

**Affiliations:** Toronto General Hospital, University Health Network

**Keywords:** supraventricular tachycardia, atrioventricular nodal re-entry tachycardia, orthodromic reciprocating tachycardia, RF ablation, cycle length variation

## Abstract

Major spontaneous variation in cycle length during supraventricular tachycardia is quite an uncommon phenomenon, which sometimes can mislead a correct diagnosis. We describe a patient who developed spontaneous variation in cycle length during electrophysiologic study in whom the coronary sinus cannulation was extremely difficult. In this situation, careful inspection of the mechanisms associated with this variation and classic pacing maneuvers are important to make a correct diagnosis of the supraventricular tachycardia.

##  Introduction

Major cycle length (CL) variations are unusual in supraventricular tachycardia. These variations can occur due to several mechanisms, which are not always obvious [1]. In patients with dual atrioventricular (AV) nodal physiology and accessory pathway, more than one circuit can be responsible for the generation and maintenance of the arrhythmia [2]. The electrophysiologic study (EPS) is a fundamental diagnostic test to help differentiate tachycardia mechanisms. We present a case of orthodromic atrioventricular tachycardia (ORT) in a patient with dual AV physiology, in which a sudden variation in CL was observed. The challenges and difficulties during EPS are reported.

## Case Report

**Figure 1:**
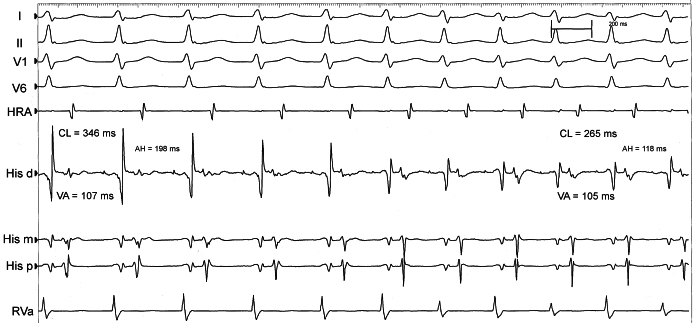
Narrow complex-tachycardia with 1:1 VA relationship and sudden change in cycle length from 346 to 265 ms. Shown are surface ECG leads I, II, V1 and V6; high right atrial electrogram (HRA); His bundle electrograms - proximal (His p), mid (His m) and distal (His d); right ventricular apex electrogram (RVA).

A 20-year-old man underwent EPS for evaluation of recurrent palpitations. Baseline 12-lead electrocardiogram was normal. Catheter positioning induced a sustained narrow QRS complex tachycardia with 1:1 ventriculo-atrial (VA) relationship. Coronary sinus (CS) cannulation was extremely difficult due to anatomical reasons and could not be performed in the beginning of the study. During the tachycardia, there was a spontaneous change in CL from 346 to 265 ms. A significant QRS voltage alternans was also observed (Figure 1). What are the possible mechanisms associated with this spontaneous change in CL? Is it possible to make a correct diagnosis of the tachycardia without the CS catheter appropriately positioned?

## Discussion

Minor CL variations are frequently seen during supraventricular tachycardia, but oscillations above 15 ms are unusual. These changes can occur by a variety of mechanisms, particularly due to autonomic influences. In a re-entrant mechanism, a shift in either the antegrade or the retrograde limb, or a change in the conduction time along these limbs can contribute to the CL change. In tachycardias due to enhanced automaticity, either a shift in the automatic focus or varying degrees of exit block from the focus can contribute to the change in CL [3-5].

Figure 1 shows a narrow complex tachycardia at a CL of 346 ms, changing to a CL of 265 ms. There is 1:1 VA association with VA interval of 105 ms. The differential diagnoses of the tachycardia include atrioventricular nodal re-entry tachycardia (AVNRT), ORT and atrial tachycardia (AT). During the change in CL, atrial CL variation is predicted by changes in the preceding ventricular CL. This makes a diagnosis of AT unlikely and supports a diagnosis of either typical (slow-fast) AVNRT or ORT. In these arrhythmias the antegrade limb of the circuit (AV node) is highly dependent on autonomic tone. Conversely, the retrograde limb (fast AV nodal pathway or concealed bypass tract, respectively) is stable during the entire tachycardia. In AT, however, variations in CL are associated with the atrium and do not depend on the AV node. In this situation, AA interval determines the subsequent VV interval [5].

**Figure 2:**
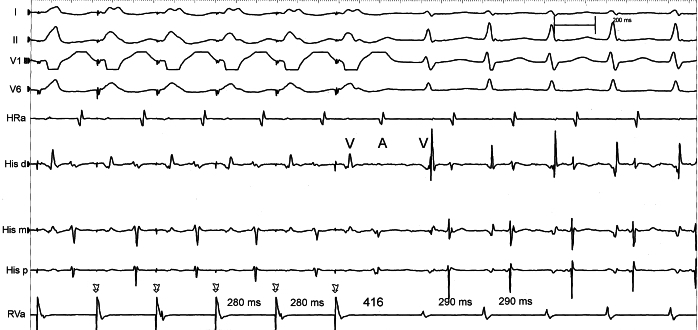
Tachycardia entrainment from RV apex. Annotations are as in figure 1. When RV pacing was stopped, the response is VAV. The corrected post pacing interval minus tachycardia cycle length is 96 ms (not shown). Arrows represent pacing stimulus.

**Figure 3:**
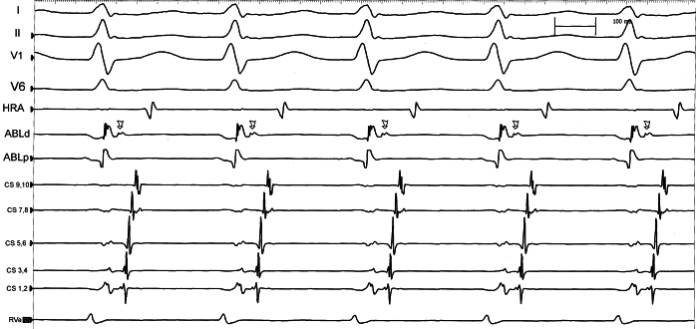
Narrow complex-tachycardia after CS cannulation.  Annotations are as in figure 1. In addition electrograms from proximal (ABLp) and distal (ABLd) ablation catheter and coronary sinus (proximal - 9,10 to distal - 1,2) are shown. The coronary sinus catheter shows eccentric atrial activation pattern. Arrows represent earliest retrograde atrial activation in the ABLd.

In the beginning of tachycardia (CL 346 ms), the AH, HA and VA intervals are 198 ms, 175 ms and 107 ms respectively. When the tachycardia CL changes (265 ms), the intervals are 118 ms, 150 ms and 105 ms respectively. So, it is possible to conclude that the change in tachycardia CL is predominantly due to change in conduction along the antegrade limb, which is in this case the AV node. Additionally, retrograde atrial activation sequence remains the same during the CL change. These findings are highly suggestive of a change in the antegrade conduction through the AV node. Overdrive ventricular pacing was done to entrain the tachycardia and the response on stopping the entrainment was VAV: further evidence against AT. The post pacing interval minus tachycardia cycle length (PPI-TCL) was 126 ms and the difference (corrected for the AV nodal delay during entrainment) was 96 ms. Also there is evidence of progressive fusion during tachycardia entrainment by ventricular pacing. These findings are in favour of a pathway as the tachycardia mechanism. (Figure 2) [6,7]. With a strong evidence of a concealed accessory pathway (AP), tricuspid annulus and right septal region were initially mapped during tachycardia. However there was no area of early atrial activation identified along these areas. Coronary sinus cannulation was reattempted and a 10-pole catheter was successfully positioned into the coronary sinus. The EP study was continued. There was evidence of dual AV nodal physiology during programmed atrial stimulation. The VA conduction during ventricular pacing was eccentric, with earliest activation in distal pole of the CS catheter, which typically occurs with left sided concealed accessory pathways. Then, a 4mm ablation catheter was positioned in the mitral annulus region via retrograde transaortic approach. Left lateral accessory pathway was successfully mapped and ablated (Figure 3). Post ablation, despite dual AV nodal physiology, there was no inducible tachycardia.

The present case illustrates an ORT using a concealed left lateral bypass in a patient with dual AV node physiology, which has been previously reported [2].  The incidence of AVNRT in patients with bypass tract is relatively common (8-10%). This may be related to the high incidence of dual AV nodal physiology observed in such patients. Also, though the presence of QRS alternans has been reported to occur more commonly with ORT, it can also occur in AVNRT [3,8].

The challenging component of this case was the significant delay in CS cannulation, which can make the diagnosis difficult. The tachycardia was successfully terminated by accessory pathway ablation. Moreover, AV nodal slow pathway ablation strategy could be considered even in patients with no inducible AVNRT in special populations (elderly patients, for example) in order to reduce the risks due to multiple EPS for recurrent arrhythmias [9].

Although the exact mechanism associated with change in tachycardia CL is unknown, it is suggestive that antegrade conduction modification through the AV node was the causal factor. AV node accommodation is possible, but not the most probable explanation for the change in CL, since a gradual increase in heart rate would be expected [5]. Thus, a shift in conduction from AV nodal slow pathway (AH=198 ms) to a fast pathway (AH=118 ms) is the most probable mechanism associated with CL in this case. In many patients, there appear to be at least two discrete inputs from the atria to the AV node, which are commonly termed fast and slow pathways. In sinus rhythm, the fast pathway constitutes the normal entry of the atrial impulse to the compact AV node. During tachycardia, the autonomic state can change the refractory properties of the slow and fast pathway resulting in shifting of conduction from one pathway to another as seen in our patient. If there is random shift in the antegrade conduction between fast and slow pathway, then the rhythm can get irregular and mimic atrial tachycardia and atrial fibrillation. This case emphasizes the importance of pacing maneuvers to help the electrophysiologist to suspect and confirm the presence of an accessory pathway, even when coronary sinus cannulation is not possible.
